# Cultural Capital as Class Strength and Gendered Educational Choices of Chinese Female Students in the United Kingdom

**DOI:** 10.3389/fpsyg.2020.584360

**Published:** 2021-01-18

**Authors:** Siqi Zhang, Xiaoqing Tang

**Affiliations:** ^1^Moray House School of Education and Sport, The University of Edinburgh, Edinburgh, United Kingdom; ^2^School of Philosophy, Zhongnan University of Economics and Law, Wuhan, China

**Keywords:** gender, class, study aspiration, international higher education, strength

## Abstract

The present qualitative study analyzes how cultural capital, gender, class, and family involvement impact Chinese female students’ aspirations of studying in the United Kingdom. We investigated how these factors facilitate or limit female students’ choice of study destination, as well as choices of subject and program. Data were gathered through participant observation and semi-structured interviews in a British university. A total of 25 young Chinese female students from different subject areas took part in the semi-structured interviews. Out of those, five students are undergraduates, 11 are taught master’s students, and the other nine students are doctoral candidates. Most of the undergraduates and postgraduates are from middle-class families, while some of the Ph.D. students are from working-class families. The results of the content analysis were examined in light of gender and cultural capital theory. It was found that although there exist differences within the middle-class families regarding the possession of cultural capital, many female students from middle-class families obtained high levels of cultural capital, and these students usually internalized the idea of pursuing a place in the United Kingdom’s tertiary education system as a way of enhancing women’s competency in future job markets. Furthermore, compared with working-class students, many respondents’ choice of subject and program was highly gendered, as their families expect them to live a feminine life by choosing “appropriate” feminine subjects. Therefore, despite having the privilege to study abroad, female middle-class students’ educational choices are still constrained by gender and class.

## Introduction

With the rapid rise in popularity of international education, more students are seeking higher education in foreign countries. For most students, studying abroad is largely regarded as a new life experience, which provides opportunities for personal development and the exploration of another country’s culture (King and Gelices, 2003). According to 2019 study, Chinese students now make up the largest and most rapidly growing group of international students in British universities (Studying-in-UK Report, 2019). With the implementation of the one-child policy, enhancing women’s standing in the Chinese family is the key to understanding the rise of transnational education among young urban middle-class Chinese women (Kajanus, 2015). Urban Chinese women, particularly those who were born after the 1980s, tend to pursue higher education abroad at an increasing rate (Center for China and Globlisation, 2015). Studying abroad, thus, became a common form of transnational mobility for young affluent middle-class Chinese women between the ages of 18 and 35 (Kajanus, 2015).

Cultural capital refers to high status cultural signals. It is a term coined by Pierre Bourdieu to analyze how culture and education contribute to social status reproduction (Lamont and Lareau, 1988). According to Bourdieu (1986), cultural capital comes in three forms—embodied (e.g., ways of acting, thinking, talking, and perceiving), objectified (e.g., cultural items such as books, instruments, and paintings), and institutionalized (e.g., qualifications and education credentials), which can be accumulated, exchanged, and transmitted. It is different from other two dimensions of capitals, i.e., economic capital, which refers to the assets that are “immediately and directly convertible into money and may be institutionalized in the form of property rights” (Bourdieu, 1986: 242), and social capital, which is seen as a property of the individual derived primarily from one’s social position and status, which enables the actor to exert power on group or individuals to position themselves (Bourdieu, 1986). Most of the research in the field of sociology of education and transnational mobility focuses on how middle-class families secure their privileged status and ensure social reproduction for their offspring. For instance, getting higher educational degrees, especially from western countries, is regarded as a kind of institutionalized cultural capital and considered an effective way to enable their offspring to get the most valued and highly sought-after occupations (Waters, 2006, 2009; Sin, 2009; Waters and Brooks, 2010; and Kim, 2011). Current literature indicates that the choices of destination, institution, and subject of study are part of a strategy ultimately aimed at maintaining social status and increasing cultural capital (Sin, 2014; Sheng, 2015; Tindal et al., 2015). For instance, students’ subject choices are usually closely aligned to specific career and “employability” objectives, such as business, management, and engineering. In terms of destination countries, the United States is the top host country for Chinese international students, while the United Kingdom normally ranks as the second most popular host country. According to the statistics of UK Council for International Students Affairs (2017), during the 8 years from 2010 to 2017, the number of Chinese students in the United Kingdom grew at an average rate of 9% annually, and China is the top one non-EU sending country of the international students. However, it is still not clear whether Chinese families believe that achieving social mobility or social status reproduction through pursuing international higher education in developed countries is an effective method for their daughters or what the specific educational choices of young middle-class Chinese women rather than Chinese students in general are.

Social class status was found to significantly influence family educational expectations and students’ study performance (Byun et al., 2012). On the one hand, parental involvement plays a crucial role in influencing an offspring’s educational choices, such as whether to pursue advanced degrees after graduation or not, when to study abroad, and the choice of potential universities (Devine, 2004; Sin, 2014). It is believed that middle-class families’ expectations of educational success, which serve as an important means of class reproduction, shape their offspring’s aspirations to achieve success in their educational and career goals (Devine, 2004). On the other hand, students from privileged families obtain more educational resources, and they tend to have higher academic achievement (Bourdieu, 1986; Byun et al., 2012; Duan et al., 2018). It should be noted that educational resources particularly cultural capital, for middle-class students, is primarily accumulated within their families. Exposure to activities such as reading, learning to play classical instruments, and going on educational visits provides middle-class children with cultural capital. There exists a positive link between the possession of certain types of cultural capital, such as the British storybooks, and middle-class children’s decision to study in the United Kingdom (Sin, 2014). These middle-class students are more likely to succeed because the early accumulation of cultural capital gives them various strengths, such as a sense of independence and self-confidence, aesthetic disposition, and modified behaviors and lifestyles (Sin, 2014; Xu, 2017). All of these are more likely to provide them with an advantage at school and future workplaces. Devine (2004) study found that a multitude of middle-class families employ their family advantages and middle-class privilege to help their offspring achieve high academic goals and to acquire decent occupations. In this way, middle-class children’s academic and occupational advantages are reproduced by middle-class families. However, Devine (2004) also suggested that middle-class families do not have possession of all the same kinds of economic capital or cultural capital and were also stratified by degrees of their access to economic, cultural, and social capital.

In terms of gender and transnational educational mobility, the current literature on international students has discussed how gender impacts international students’ mobility. For example, Kajanus (2015) research shows that young urban Chinese women have obtained the greatest power to get sufficient educational resource from families in their role as daughters. Most of the students and returnees in the study are from urban cities with only a few participants from rural areas of China. Many young Chinese women pursue gender equality before studying abroad and dare to challenge Chinese gender norms during educational mobility. Bamber (2014) study suggested that although it is quite common to see more Chinese female students participating in the transnational educational flow, gender equality or freedom remains one of the significant aspirations of these students. In order to seek what motivated Asian female students to study abroad, Kim (2010) addresses the function of media in motivating female students’ aspirations to participate in the educational migration process. Through interviewing Asian women from Japan, China, and Korea who aspired to study abroad, Kim (2010) argues that these young women’s imaginary travel from western media generates a rising individualization for female students and influences women’s attitudes toward femininity. In Sin (2014) research, western media is interpreted as a form of cultural capital for international Asian students. Female individualization operates as “a self-reflexive and social practice” through engagement with Western media in their homeland before they move (Kim, 2010, p. 25).

Most of the current literature positively considers Chinese female students as privileged women (Martin, 2016). Pursuing higher education abroad can help female students to be independent and to be more competitive in future job markets. However, the traditional gender norms of getting married at certain ages and being mothers/wives might continuously exert influence to different extents or shape their perceptions through transnational ties they established with parents and relatives during their studies, which still restrict their future career and life choices (Kajanus, 2015). Female students’ choices of subjects are still gendered, and these made their future career choices gendered again (Sheng, 2015). In traditional Confucian thought, women were subordinate to men, which has exerted long-lasting influence in Chinese society, while in the post-reform China, such belief has been changed, gender equality has been promoted, and many Chinese women’s participation in paid labor market improved their status markedly (Hughes and Maurer-Fazio, 2002). However, with the fierce competition in the workplace and increasing life expenses in urban China (i.e., the skyrocketing housing prices and children’s education), the gender view that “doing well is inferior to marrying well” becomes increasingly popular in Chinese (Zhao et al., 2019).

Currently, research regarding how gender and the possession of different types of cultural capital within middle-class Chinese families can impact their offspring’s educational aspirations is still lacking. Therefore, it is important to explore how the interplay of gender, class, and family involvement impacts the aspirations of Chinese female students who come from different social class backgrounds. In order to address the gap existing in current literature, this study proposes the following research question: How do family cultural capital and gender construct Chinese women’s educational aspirations (particularly decisions on study destination, subject, and programs) before their travel? We will define the key concepts used in this paper. This paper begins by exploring the relationship between the accumulation of cultural capital in middle-class families and students’ aspirations of studying in United Kingdom. After examining how their families transmit cultural capital to them, we will look at how gender, age, and class facilitate or limit female students’ decisions when choosing subjects and programs as anticipated and experienced by the participants.

### Conceptual Framework

The middle class in China is emerging along with the rapid modernization and urbanization of the country (Xiao, 2001; Lu, 2002). Due to the reform and the opening-up policy, starting in the 1990s, private entrepreneurs as well as self-employed individual businesses experienced rapid growth and became the new middle class. Li C. (2011), Li M. (2011) argues that many Chinese do not fall into easily identifiable class divisions. Among many resources and standards to identify social stratification, income, occupation, and education are three indicators commonly used for measuring individual socioeconomic status (Li and Zhang, 2008). The middle class in China involves those who occupy white-collar jobs, hold middle and advanced educational degrees, and possess wealth on and above the average level, and they are mainly in urban areas. Generally, according to the two sources of “redistribution and market” for producing middle class in contemporary China, there is a typified distinction of middle-class in China: *inside the system*, which refers to those who are beneficial, occupying jobs as governmental officials, public school and university teachers, and doctors as well as the managerial staff in state-owned enterprises: and *outside the system*, which refers to entrepreneurs and managerial staff in non-state-owned and foreign enterprises or business (Wang, 2004). Comparatively, those *inside the system* are the intellectual and well-educated and hold more official power or resources in political or institutional system, while those *outside the system* are the wealthy and are mostly educated; both of them have the motivation and capability to get access to better education and resources in society. As a matter of fact, even within the middle-class Chinese system, the upper middle-class inside system and lower middle-class system have accumulated different levels of social and cultural capital (Chen, 2013). Besides, industrial service workers, laid-off workers, and rural–urban migrant workers are generally regarded as the Chinese working class. The possession of different levels of cultural capital influences middle-class families’ educational aspirations. In this study, middle-class students are from both inside system backgrounds and outside system backgrounds.

Cultural capital functions as status markers, conferring individuals who possesses cultural capital with superior power to legitimate their values, tastes, and cultural practices in a certain site (Bourdieu, 1984). The “students’ educational aspiration” in this study is shaped by their parental institutionalized cultural capital, and the objectified cultural capital and embodied cultural capital in their families. All these cultural capitals facilitate students’ internationalization of family educational aspirations. Among these three forms of cultural capital, we focus more on the embodied form of cultural capital. Bourdieu(1977, p. 47) conceptualizes embodied cultural capital as “long-lasting dispositions of the mind and body.” The long-lasting embodied cultural capital students possessed particularly the gendered disposition of mind that is directly related to the class-based families. Although students may have their own individual aspirations and own agency to go against the family aspiration, this research addresses more on the issue of how their internalized habitus, especially family expectation influences their education choices; thus, we tend to address the data from the students’ own belief and thoughts, which are regarded as the reflection of their embodied class-based family cultural capital.

Gender in this research is understood as complicated social relations and involves social activities, including students’ understanding of how gender is related to their educational choices. Whether gender norms such as getting married at a certain age, a desirable woman is a good daughter, virtuous wife, and selfless mother, as well as “doing well is inferior to marrying well” mentioned before, have exerted influence on Chinese female students is rarely known. To be specific, the gendered practices in this research focus more on how gendered expectation, including the marriage expectation and educational expectation, impacts female students’ educational choices, such as their decisions of choosing subjects, programs, and study destination.

## Materials and Methods

### Participants

In order to address the research questions, a British university was chosen as a site of investigation. From the 2018 Student Body by Level of Study and Gender Report from this university where the researchers conducted this research, female students outnumber the male students in the total number of all the students who are studying in this university. This university attracts high numbers of students from China, so we consider that it might be one of the proper cases to show Chinese female students’ educational choices.

Purposive sampling was employed to conduct a semi-structure interview because this research has a special target. This research relates to international student migrants (King, 2002); it requires international students to have already resided in the host country for not less than 1 year. So visiting students, exchange students, or scholars were not included in this project. However, the 1-year taught master’s students were still involved in this research because their program is equal to 1 year, and according to their visa, they can reside in the host country for 16 months. It was ensured that the participants were women from Mainland China in the 18–35 age group. There are 25 interviewees who came from different backgrounds, including diverse study subjects, birthplaces, ages, educational levels, and social classes. Among the semi-structured interview respondents, five students are undergraduates, 11 students are taught master’s, and the other nine are Ph.D. students. Twenty-one students are from middle-class families, while four Ph.D. students are from working-class families. All the respondents are from a broad array of disciplines ranging from engineering and sciences to humanities and social sciences. Table 1 presents participants’ names (pseudonyms) in semi-structured interviews and main characteristics, such as subjects and programs. Table 2 presents the information on the informal interview from participant observation with participants’ names (pseudonyms). The respondents were recruited with an advertisement posted in WeChat groups, student accommodations, and students whom the researchers interacted with daily.

**TABLE 1:** Profile of semi-structured interview cases.

Pseudonym	Subjects	Programs	Class
Jianeng	Science and engineering	Ph.D.	Working class
Mingjing	Arts, humanities, and social sciences	Undergraduate	Middle class
Yami	Science and engineering	Ph.D.	Working class
Xiaolin	Arts, humanities, and social sciences	Master	Middle class
Luyi	Science and engineering	Ph.D.	Working class
Lucy	Arts, humanities, and social sciences	Master	Middle class
Jennifer	Arts, humanities, and social sciences	Undergraduates	Middle class
Mianjia	Science and engineering	Ph.D.	Middle class
Coco	Arts, humanities, and social sciences	Master	Middle class
Lilan	Arts, humanities, and social sciences	Undergraduates	Middle class
Wenyu	Arts, humanities, and social sciences	Master	Middle class
Feifei	Arts, humanities, and social sciences	Undergraduates	Middle class
Licui	Arts, humanities, and social sciences	Master	Middle class
Wangqi	Arts, humanities, and social sciences	Undergraduates	Middle class
Lingpin	Science and engineering	Master	Middle class
Liyu	Arts, humanities, and social sciences	Master	Middle class
Tianrun	Arts, humanities, and social sciences	Ph.D.	Middle class
Lulin	Arts, humanities, and social sciences	Master	Middle class
Louise	Arts, humanities, and social sciences	Ph.D.	Middle class
Yilian	Arts, humanities, and social sciences	Ph.D.	Middle class
Mingyu	Science and engineering	Master	Middle class
Lidan	Science and engineering	Ph.D.	Middle class
Monica	Science and engineering	Master	Middle class
Jiahui	Arts, humanities, and social sciences	Master	Middle class
Pingping	Science and engineering	Ph.D.	Working class

**TABLE 2:** Profile of informal interview cases from participant observation.

Pseudonym	Subjects	Programs	Class
Mimo	Science and engineering	Master	Working class
Ying	Arts, humanities, and social sciences	Ph.D.	Middle class
Lingxiao	Science and engineering	Ph.D.	Working class
Linchang	Arts, humanities, and social sciences	Ph.D.	Working class
Yili	Arts, humanities, and social sciences	Master	Middle class
Sue	Arts, humanities, and social sciences	Master	Middle class
Fenfen	Arts, humanities, and social sciences	Master	Middle class
Wupin	Arts, humanities, and social sciences	Master	Middle class
Jade	Science and engineering	Master	Middle class

### Data Collection

The mixed qualitative methods of participant observation and semi-structured interviews were conducted, which allowed for the exploration of individual and personal attitudes and beliefs, to generate an in-depth account of the gendered and classed aspirations of Chinese female students in this research. Observations took place throughout the whole research process. The formal duration of the fieldwork was from November 2015 to November 2016. Interviews took place in their preferred places such as their study campus, their accommodations, a meeting room in the library, in their departments, and a café near the campus. Sometimes, interviews and participant observations overlapped due to the fact that the mutual conversation, in and of itself, provides an important amount of data for both the methods. To identify the participants’ family social class backgrounds, firstly, information about three commonly used objective indicators was collected during the interviews, i.e., their parents’ occupations, educational levels, and annual family income. Meanwhile, in the participant observation, the researchers observed participants’ family background through their daily interaction, such as their lifestyles, clothes, and consumption ability as well as past experiences of education, travels, and other culture and leisure activities; then their observations were confirmed by the social class information of the participants. The data presented in this research are from both a formal interview and participant observation. A formal interview in this research is defined as a mutual 1-h conversation between one respondent and one researcher only, with formal consent from the respondents, held a quiet place. Other kinds of conversations, those less than 30 min and are referred to as informal interviews in participant observation during daily interaction with participants, were also included in this study. Participants were informed in advance that they could speak either English or Mandarin Chinese in their daily conversations or formal interviews. Open-ended questions were used during interviews, which allowed flexibility to explore in-depth responses (Knox and Burkard, 2009). Respondents were informed that the whole interview would be recorded for the use of transcription. Participants were also informed that they were free to stop the conversation at any point if they felt uncomfortable in both semi-structured interviews and participant observation. It was guaranteed that all the respondents understood the aim of this research and agreed to be participants. In semi-structured interviews, all participants signed the consent form. In participant observation, on some occasions, especially in the big public activities organized by the student leaders, the researchers informed the student leaders. When it comes to a private occasion such as a private party of limited people, researchers gave the research information to participants.

In qualitative research, researchers also have certain assumptions, beliefs, and values. Gender and class influence researchers’ position (McCorkel and Myers, 2003). Researchers’ positionality plays a vital role in how they conduct fieldwork, how they select useful data, and what they obtained from the research field (Chiseri-Strater, 1996). The researchers’ positionality in this research is considered an insider, as the researchers, when this research was conducted, were also middle-class Chinese female international students. By sharing a similar experience of studying abroad with informants, the cultural background enabled researchers to establish rapport with respondents. The disadvantage of being an insider is that it is easy for researchers to blur the boundaries of being respondents’ friends and being researchers. Thus, the researchers kept a balance of the role as both an insider and a female student migrant, avoided blurring the roles, and minimized personal bias as much as possible.

### Data Analysis

All the interviewees preferred to use Mandarin Chinese, with the occasional insertion of an English phrase to capture an exact meaning. In order to ensure the coherence of the conversation and sort out the useful data, all the data taken during researchers’ fieldwork were in Chinese. The translations were done by the first author in accord with her understanding of respondents’ narrations. At the very beginning of the translation and transcription process, researchers attempted to transcribe and translate all data in the formal or informal interviews at the same time. However, due to the time limit, researchers transcribed all the data in Chinese. Looking at the Chinese transcripts, different themes and codes were identified according to respondents’ narration. Thematic analysis was used as an analytical tool, which captures certain themes that emerged from the data (Flick, 2015). The three defined themes are as follows: female students’ study destination, class and gender constraint for their educational mobility, and family involvement.

The data from informal/formal interviews and participant observation were analyzed by using color coding. In this way, it is very easy for the researchers to identify which part of the interview texts or particular responses belong to which themes (Stottok et al., 2011). Codes, in this research, are keywords or short sentences related to each certain theme. Certain codes were chosen and were categorized under the three themes mentioned above in response to the research questions that we proposed. Different codes under the three themes were marked with three different colors. For example, according to the certain themes, the codes in the theme of “family involvement” are as follows: considering the length of study, choosing feminine jobs, and understanding of marriage and career/job. The color for these codes that appeared in the participants’ responses are all marked in red. Then, having on hand the classified color codes, researchers selectively translated the useful data into English in the places needed for analysis.

## Results

### Cultural Capital and Female Students’ Study Destination Country

Middle-class families are more likely to focus on their offspring’s educational achievements by transmitting high levels of cultural capital inside the family before students move abroad. The objectified cultural capital that Chinese female students’ possessed motivates their aspiration of pursuing higher educational degrees in the United Kingdom. In terms of the destination country for doing a degree, most of the respondents’ decisions largely relied on Britain’s long-lasting cultural impact on the media. As participant Wenyu claimed:

From primary school to university, I kept reading different English novels as there are a lot books in my home. I also watched many English films. My father obtained his Master degree in the United Kingdom … he always shared his experience. When I was young, I decided I wanted to study there.

According to her narrations above, watching films, videos, and reading novels related to United Kingdom’s culture can be seen as a vital accumulation of objectified cultural capital before her move, which also facilitated a strong desire to participate in the United Kingdom’s cultural events. Meanwhile, the parents’ institutionalized cultural capital (i.e., academic credentials or professional qualifications) has an influence on these students’ aspirations of studying in the United Kingdom. The common denominator among this group of participants who possess high levels of cultural capital is that they share similar family backgrounds; most of their parents are part of the middle-class inside system, such as university (professors), school (teachers), and government officers (public server); or they study similar subjects in the humanities and arts.

For some of the students who come from the middle-class outside system families, these students do not obtain much cultural capital, but the high level of economic capital they possessed can actually convert to embodied cultural capital as well (such as changing lifestyle) or enable them to consume material goods. For example, Fenfen was born into a middle-class family outside the system, and her parents are business people in China. It seems that when she was learning English, she read few British novels, but her reason for reading was just to learn a language:

Apart from studying, the reason I am coming here is that … I suppose that the United Kingdom is a shopping paradise with many international brands and sales in winter and summer holidays … after coming here, I think one of the big changes I have is that I quite often go to gyms because my western friends always go to gym.

As can be seen, Fenfen was born into a family with a great deal of economic capital, so her desire for material consumption is quite strong, while those whose parents are inside the system (university professors, teachers, governmental officials, and public servants) preferred cultural consumption before and during their stay in the United Kingdom. Middle-class families might possess different forms of cultural capital: some possess more economic capital, while others possess more cultural capital. The economic capital that Fenfen possessed made it easier for her to consume material goods in the United Kingdom. Also, her transnational life in the United Kingdom changed her lifestyle and behavior: going to the gym can be seen as embodied cultural capital.

After arriving in the United Kingdom, most students from families of the middle-class inside system and some students from families of the middle-class outside system accumulated more embodied cultural capital through participating in cultural activities and consuming cultural goods, which is considered by them as a means to enhance their competitiveness in job and marriage market and further secure their middle-class social status. From respondents’ perspectives, especially for those who had accumulated high levels of cultural capital before going abroad, their study destination enables them to participate in various cultural events, which facilitates further accumulation of cultural capital abroad.

Participant Mingjing, an undergraduate humanities student, was reluctant to go to countries like Australia, Canada, or New Zealand because she believed that these are countries with a very short history as compared with the United Kingdom, which is a place where she can fulfill her need of “absorbing a fruitful culture.” According to her description:

The meaning of studying abroad is not only to get a degree, but also absorbing a fruitful culture in its country such as going to its local museums, etc. I will be more competitive if I have more knowledge about the United Kingdom and the world when finding my preferred jobs, which need more cultural knowledge.

Like Mingjing, many female students often go to local museums, art galleries, and theaters in their spare time. A testimonial from participant Yili, a student whose parents are business people working in a foreign-owned enterprise, supports this:

I often go to Blackwell’s bookshop to buy books and attend local book launches. I think going to local museums, art galleries, theaters and book launches gives women natural elegance and confidence. I suppose this make me more attractive to potential proper partner who are also from middle-class families.

Attending new local book launches is also a kind of cultural event. Compared with the case of participant Fenfen, this indicates that not all students from families of business people are reluctant to participate in cultural activities. The similarity of Mingjing and Yili’s cases is that they both attach great importance to the embodied cultural capital and regard it as important strength to enhance their competitiveness in future job and marriage markets, which further facilitates middle-class status reproduction.

Apart from that, few female students are from working-class families. Aspirations and experiences of these students are different from those of their middle-class counterparts, and they generally possess less cultural capital. For instance, participant Lingxiao revealed that:

I did not go to cultural activities before I came to United Kingdom. When I came here, I don’t do this either because I think this might not be directly useful to find a decent job. But a good degree with high scores will be. So I work very hard … I think high scores and a degree with distinction will help me to find a good job.

For working-class students, getting international institutionalized cultural capital, particularly good degrees and high scores, seems to be more significant for them in order to achieve upward social mobility. The cases above clearly show that a respondent’s focus and choice of a certain destination country are largely influenced by the capital (either economic capital or cultural capital) owned by the family. Also, there exists a level of complexity within different class-based families. Even for middle-class families, the capital that students obtained is quite diverse.

### Class and Gender Constraint for Educational Mobility

Chinese female students’ choices related to studying abroad are still complex and constrained by their age, gender, and class, especially when they are considering what kinds of programs to study and when to study abroad. Most respondents revealed that their families required them to pursue a degree abroad at an appropriate age (especially not over the age of 27) if they are still single. Being single over the age of 27 designates a woman as a “leftover woman” in China’s dominant cultural norms (Nakano, 2014). This means that once a respondent’s age is over the so-called age limit, they have to make greater efforts to persuade their family to support them. In terms of social class background, respondents, particularly from lower-class or lower-middle-class families, sometimes even had to make huge sacrifices to realize their dreams of seeking an overseas education.

For example, participant Licui, a single female master’s degree student revealed:

My parents hesitated to support me to study abroad when I was 29. Considering a taught master’s in Britain only cost 1 year, they finally let me (study abroad). They were against my decision to do Ph.D. as it is hard to find an appropriate partner in China afterward.

Apart from her age, being born into a lower middle-class family made it more difficult for her to afford the large expense of the overseas tuition fee for a 4-year Ph.D. program:

I got my Ph.D. offer but I did not apply for a scholarship successfully. If I stick to my choice, my family said that they would have to sell their house in the city in order to get sufficient money for the 4 years of study.

From Licui’s narration, the lack of sufficient economic capital to support overseas higher-education pursuits is a prominent issue. Age is another important issue in influencing her decision to study abroad. The central logic of this phenomenon is that single women face earlier marriage deadlines. Participant Xiaolin, a student from an affluent middle-class family also indicated that:

Most of female students study abroad from 22 to 25. My family told me their support was conditional: I should go for it before 25, otherwise, I will lose their support.

Doing a 1-year taught master’s degree in Britain indeed saves these respondents’ time. Like them, most of our respondents who are around 27 years old expressed conflicting feelings over their studies since getting an advanced degree could enhance their job competency, but they still have to worry (if they spent a year or two in transnational study) about being less competitive in the Chinese marriage market.

However, interestingly, a few respondents from working-class families revealed that Chinese marriage gender norms are not always disadvantageous for them when making decisions about pursuing higher education abroad. With the privatization of the housing market in China, a new trend emerged. It is expected by the bride (or her family) that the groom should own a flat in preparation for their marriage (To, 2013). This is a relatively new gender norm, which increases men’s burden but relieves pressure on women to prepare flats for marriage. Mimo, a 22-year-old master’s student, recounted:

My family is not rich….I think they support me studying because they do not have the pressure to buy a flat in preparation of a marriage after my return, so they can invest all their money in my 1-year master’s education.

Mimo’s parents are industrial workers in the southern part of China. Their parents had spent their entire life savings to support her 1-year study in the United Kingdom. Mimo’s example shows that, for the families with limited economic capital, the relatively new gender norm, which requires Chinese men to buy a flat before marriage, does not always have a negative effect on families’ decisions to support their daughters in studying abroad, especially when they are young.

Although respondents’ exact choices of programs and study duration are constrained by gender, age, and class, some respondents still regard overseas education in the United Kingdom as a positive and useful means to fight against gender discrimination in China’s labor market. For example, Jade, a 30-year-old student, holds the belief that studying abroad will increase her competitiveness in the job market. Before she studied abroad, she was a doctor working in a state hospital with a limited salary, but she aspired to transfer to a private hospital, which might offer her a better salary. According to her accounts, private hospitals require female employees to have English fluency and an overseas background, so she decided to pursue an overseas qualification to enhance her competitiveness in order to transfer to a more lucrative job.

Moreover, as for Ph.D. students, their awareness of needing to be more outstanding than their male peers is also related to the gender discrimination in China’s job market. Tianrun, a Ph.D. student in the arts, also pointed out that:

My mother told me that because I am a woman I should be more outstanding. That is why I was determined to pursue a Ph.D. degree. But I still need to be hard-working. A female Ph.D. job-hunter must be far more outstanding than her male counterpart.

These respondents’ stories show that women should be more distinguished than male counterparts in order to get more opportunities, either in finding a good job or for future progress. It is expected that an overseas education in the United Kingdom will greatly enhance their job competitiveness in China’s labor market.

### Family Involvement and Gendered Choice of Subjects

Subject choices for overseas study were also found to be related to gender and social class. Middle-class respondents normally attach more significance to either the success of their marriage or to striking a balance between their future career and marriage. However, working-class female respondents and/or their families do not concern themselves too much with gender-related issues when choosing subjects for overseas study because studying practical subjects that enhance competitiveness in the job market and enables their possible upward mobility, is their primary concern.

Chinese female students from middle-class families are more likely to be found in subjects such as arts, business, humanities, and social sciences. Informants from middle-class families choosing subjects of hard sciences were less numerous. For example, participant Louise, a Ph.D. student in humanities, did well in chemistry when she was young and showed an interest in chemical engineering. As she revealed:

My father is a higher-degree holder and my family, especially my father, wanted me to study subjects suitable for girls, it is definitely not a good idea to learn subjects like engineering … They would like me to choose a subject which is easy to learn.

Louise’s example is a typical example, as other respondents in this study convey similar sentiments. As is shown by her excerpts, although Louise can pursue a master’s degree or even a Ph.D. degree abroad, the available choice of subjects is still gendered. This means that, even if she is a high-degree holder, she was still cultivated to be an elite only in a field that is considered feminine and socially acceptable. Thus, her family encouraged her to study a stereotypically feminine subject, as they hoped that she would be able to get an easy job as well as avoid the fierce competition of the male-dominated hard science subjects. It was interesting to find that even if Louise’s family members were high-degree holders, they still held strong gender-biased attitudes toward different subjects.

Louise’s story also showed that her father believes that he possessed a high level of cultural capital, so he himself feels that he is superior and more knowledgeable than other family members. Then during the decision-making process, he helps his daughter choose a subject that might direct her to a prospective “appropriate” feminine job. However, at the time when Louise was interviewed, she expressed her regret over not pursuing a science subject.

It is obvious that some respondents from middle-class families still regarded following a traditional feminine path, especially marriage, as equally important as their academic achievements. For instance, participant Lidan, a Ph.D. student, also indicated that:

My intention to be a Ph.D. student is quite simple: to be outstanding. But my family expects me to get an academic job because such jobs have summer/winter vacations…it strikes a balance between my future career and family once I have obtained a Ph.D. degree and gotten married.

Besides, according to some middle-class participants’ narrations, their families strongly emphasized that their happiness was related to marriage, which is also a kind of personal achievement. Although these respondents’ families supported their daughters’ decisions to study abroad, they still hoped their daughters would live a happy and relaxed life by following a traditional feminine path of having a good marriage instead of working too hard. In contrast to many participants, Sue believed that:

As long as I can obtain my degree, my parents did not require me to study extremely hard … making a good marriage is more important for women. I also wish that I can have a happy marriage, instead of working hard in a famous company.

It seems to her that she has already internalized the typical feminine ideal, which suggests that working hard does not lead to a successful life for women. However, unlike Sue, another participant, Wupin, presents a complicated view:

I wanted to have my personal achievement in my future career. After I was 25, my family suddenly began to address the importance of marriage…. Maybe in the future I will try to strike a balance between my career and my marriage.

Wupin’s perception of “striking a balance between career and marriage” is more common with most of the respondents.

As can be seen from the different examples, respondents from middle-class families are encouraged by their families to pursue a higher degree such as a master’s or a Ph.D., However, the available choices for them are often highly restricted by gender-stereotyped views as shown from their decisions on which subject areas to study and their future plans.

### Hierarchies in Programs, Subjects, and Gendered Choices

There exist hierarchies within the choice of subject among female students from different social classes. To some extent, subject and program itself can serve as a form of symbolic capital to distinguish students’ social status. In general, in terms of the three different types of programs (undergraduate, taught masters, and Ph.D.), students’ decisions to pursue particular kinds of programs before studying abroad also reflect that family social status has its own set of hierarchies. Such differences can be particularly obvious when comparing undergraduates with Ph.D. students sponsored by scholarships.

For instance, participant Lilan, an undergraduate student whose father is a president of a famous supermarket chain, revealed that:

I did not do well in my studies. My family suggested to me that studying a bachelor’s degree abroad might be a more effective solution to escape the fierce college entrance examination. The undergraduate programs here are all self-funded.

While the undergraduate’s statement claims that getting accepted into a bachelor’s degree abroad is far easier than obtaining a good national offer at home, Ph.D. students generally reveal that getting enrolled in a Ph.D. program, especially with a scholarship in Britain, seems to be much harder than getting a national Ph.D. offer from a Chinese university. It is normally acknowledged by respondents that only very outstanding students can get a Ph.D. offer in Britain together with Britain’s local scholarship. Among nine interviewees who are Ph.D. students, four students got Ph.D. offers with scholarship, and three of them were from working-class families. It is more likely that most of the outstanding Ph.D. students come from working-class families in this research. As Jianeng, a second-year physics Ph.D., point outs:

If I did not have a full scholarship, I would never have the chance to do a Ph.D. abroad. I am determined to do a Ph.D. because this will change my destiny. I keep reminding myself to be more excellent than others.

The two opposite examples above show that the choice to do a bachelor’s degree or a Ph.D. abroad is a class-based choice.

There may be many factors that motivate Chinese female Ph.D. students from working-class families to study in the United Kingdom, such as their personal interest in their studies. But some of the Ph.D. students in this research, like Jianeng, strongly desire to achieve upward social mobility through the possession of a Ph.D. degree from abroad. However, it should be noted that not all the outstanding Ph.D. students are from working-class families; there also exist some very hardworking and outstanding Ph.D. students from affluent middle-class families in this study. A majority of affluent Ph.D. students are self-funded and pursuing programs in the arts, social sciences, and humanities. When sharing the reasons why these Ph.D. students are willing to pursue self-funded degree, participant Ying revealed:

My family believes that social sciences and humanities are good subject choices for me. It is hard to apply for funding successfully in United Kingdom universities, but pursuing a Ph.D. degree will be definitely advantageous for me in finding a decent job, such as working in a university.

Meanwhile, with regard to the choice of subjects, choosing feminine subjects, especially finance and management, are regarded as the privilege of wealthy middle-class families. It seems to some participants that a large number of students in these feminine subjects are from affluent family backgrounds. As Mianjia, a middle-class engineering Ph.D. student revealed:

My classmates were from very good family backgrounds…but only two female students chose to learn engineering. There are only two reasons: either because their family members’ occupations are in the field of science or their family backgrounds is not well off.

Indeed, we noticed that although there are still respondents from middle-class families who choose subjects such as biology, chemistry, physics, and engineering, most of these respondents’ family members are also working in related science fields. In Mianjia’s case, one thing is clear: regardless of whether she is interested in science or not, her consideration is that studying a subject in the field where her family member is already established as a “safe” choice for her. Therefore, she can take advantage of the social capital (i.e., potential social network) provided by her family in finding a job after graduation. There are few other cases like Mianjia’s case; we find that all of these students’ family members are working in the engineering field regardless of middle-class inside system background or middle-class outside system background, while working-class respondents like Jianeng did not make gender-stereotypical choices because their primary concern is to gain upward social mobility through studying a practical subject in a tertiary education system in Britain.

## Discussion

This study has introduced a complicated interplay of cultural capital, gender, class, and family involvement, which all play a role in influencing Chinese women’s aspirations to study in the United Kingdom. It reveals that cultural capital in middle-class families facilitates students’ aspirations to study abroad and that middle-class reproduction makes the idea of pursuing a place in the United Kingdom’s tertiary education system more likely to be internalized. Most respondents’ aspirations for an overseas educational experience are also cultivated from their middle-class family habitus. According to Bourdieu (1977), habitus, which reflects a set of behaviors and values, acts as embodied perception and actions based on an individual’s social position in a society. Middle-class habitus is shown from the parents’ educational levels, the respondents’ media consumption, and in how the cultural capital accumulated in a family instigates the respondents’ desire to study in Britain. Female students are motivated to pursue transnational educational mobility because they are inspired by the imaginary transnational world presented by western media (Kim, 2010). It is clear that there exists a degree of complexity even within middle-class families, for those who come from both the middle-class inside system and middle-class outside system. This finding is supported by Bourdieu’s theory that the composition and numbers of the exact cultural capital in the middle class differ: for some businessmen who are from the middle-class outside system, they might have higher levels of economic capital but lack sufficient cultural capital to transmit it to their offspring (Bourdieu, 1986).

In terms of gender dimension, it is true that a driving force for female transnational mobility is their desire to get a higher qualification, which would make them distinct in comparison with male graduates in the labor market (Waters, 2009; Holloway et al., 2012). Many employers are unwilling to take the “risk” of hiring women in their twenties who, it is assumed, will soon marry and bear children, requiring them to care for a family instead of devoting all their time to a job (Holloway et al., 2012). In Kim (2011) study, Korean international students in the United States indicated that the main motivation for female Korean students to study abroad is that they aspire to be outstanding by obtaining a United States higher degree and hate Korea’s patriarchal system, which leads to a phenomenon where female faculty members are always fewer and academic events were structured around males. Our findings are also in line with Bamber (2014) research, which determined that gender discrimination motivates young women’s desire to be more outstanding by obtaining overseas qualification in order to be more competitive and secure in the fierce competition of today’s job market.

Furthermore, it is notable that female students’ actual educational choices when pursuing transnational studies (such as the study destination, programs, and subjects) are still constrained by Chinese gender norms and family class background. International Chinese female students’ motivations for studying abroad also show that Chinese female students in the United Kingdom expressed great concerns about the length of time required to complete their studies (Bamber, 2014). It is likely that the aspiration of middle-class families is particularly gendered, so they tend to advise their daughters to choose “appropriate” subjects (i.e., humanities and arts) to construct their gender identities (Sheng, 2015; Tu, 2016). As McCall (1992) argues, in the socialization process, an individual internalizes appropriate gender behavior and values. According to Bourdieu (1998) views, it is just such gendered habitus of middle-class families that make parents’ judgments valid and weigh heavily when influencing their offspring’s decision making. The dominant conventional femininity, which emphasizes making appropriately feminine career and life choices, plays a crucial role in shaping respondents’ views regarding programs of study and even the future divisions of labor. Thus, our research expands the literature made by some feminists who link gender with class (Skeggs, 1997; Adkins and Skeggs, 2005).

Middle-class students’ possession of a variety of privileged educational resources and their participation in international higher education in the United Kingdom further help them to accumulate cultural capital, which in return reproduces middle-class social status. This will not be the same in working-class families, as they do not have much cultural and economic capital. Therefore, working-class families need to pay attention to the unequal education resources obtained in families, which might lead to further unequal social class reproduction. Schools and universities could promote education equality by expanding the number of prospective students who come from working-class families and by allowing more opportunities for working-class students’ self-development and encouraging them to participate in cultural events.

Middle-class Chinese young women have accumulated cultural capital in their families, which show various strength and potentials. Studying abroad in the United Kingdom helps them accumulate cultural capital, and students believe that this enhances their competitiveness in both job and marriage markets. It should be noted that in the process of choosing study subjects, considering the background of social class, student’s personal interests, and ages, young female students could identify their own strengths and weaknesses. At the same time, it will be helpful for their parents to maintain an open mind, respecting their offspring’s choices and personal interests. Some families tend to follow the trend of choosing popular subjects and hold strong gender-stereotyped ideas related to their daughters’ studies. These families may put more pressure on their offspring if they are not interested in following the socially/familial accepted study choices. More mutual communication, discussion, and support are needed to ensure that parents and their offspring fully understand each other and make the most suitable study choices.

Among all the female students, there are some young working-class women who ambitiously attempt to change their destiny and enhance their job competitiveness through seeking higher education abroad. They are more likely to choose practical and realistic subjects and pay less attention to their ages or not hold gender-stereotyped views. Taking the practical approach of studying a science subject might offer working-class students more opportunities in the labor market (Sheng, 2015). For these groups of students, in order to achieve upward mobility, pursuing higher education abroad is a great opportunity for them to acquire the cultural capital that comes from getting higher educational degrees from western countries and learning knowledge or skills, which will be useful in job markets.

### Limitations and Future Directions

The present study has some limitations. Firstly, participants in this research are only from one British university, and the number of participants is limited, so the application of these findings to other Chinese students who study in other United Kingdom universities is inadvisable. In the future, students from different institutions in other places of Britain (England, Wales, or Northern Ireland) might add more value to the current study. Besides, although our research presents the complexity of the students among different groups (such as ages, programs, or subjects), most of them are middle-class students. The study aspirations of working-class students who study abroad should also receive attention. Compared with middle-class female students’ educational choices of studying abroad, the working-class female students tend to choose practical subjects. Moreover, female middle-class students’ intentions of social class reproduction and working-class women might also achieve upward mobility from a transnational study experience. It is not very clear whether there will be complexity within the group of working-class students, and it is still important to include more working-class students and have more comparisons of the educational choices between working-class and middle-class students. Therefore, another limitation that needs to be solved is that is in this study, we identify participants’ social class based on the limited information (such as their family income, parents’ educational attainment, and occupations) reported by each participant and observations. In future studies, quantitative research with accurate socioeconomic status measures (both objective and subjective indicators), strict sampling procedure, and appropriate sampling size should be conducted to strengthen the representation of the participants and deepen our understanding of Chinese female international students.

Finally, the inclusion of participants’ family members, such as parents, could be informative in casting another perspective on the topic of how they influence students’ transnational study choices from their perspectives. This research has attached great importance to how respondents’ families influence them to accumulate cultural capital before they study abroad. However, all of the data related to respondents’ family and parents are from students’ own narrations instead of from their parents’ direct narration. Further research might also include the responses from international students’ parents as another research sample group for the purpose of reflecting on their transnational educational aspirations.

In summary, this research shows that young Chinese female students’ study choices in the United Kingdom are influenced by social class and cultural capital and still constrained by gender, class, and family involvement. It has uncovered fascinating insights and hopefully lays the groundwork for more detailed studies in the field of gender, sociology of education, and student transnational mobility. Influenced by a number of factors (such as ages and Chinese gender norms), female middle-class students’ choice of subject tends to remain along the lines of socially appropriate subjects. It has been revealed that educational mobility should be understood as a complex trajectory rather than being a symbol of simple positive transition in female students’ lives. Studying these “appropriate subjects” might potentially give these young women the push needed to get into restricted fields and eventually enter into the limited future job market.

## Data Availability Statement

The raw data supporting the conclusions of this article will be made available by the authors, without undue reservation.

## Ethics Statement

The studies involving human participants were reviewed and approved by the University of Edinburgh. The patients/participants provided their written informed consent to participate in this study.

## Author Contributions

SZ designed, conducted, and coordinated the realization of the study, and drafted the manuscript. XT revised the manuscript and made insightful comments with this article. Both authors contributed to the development of the article, manuscript revision, read, and approved the submitted version.

## Conflict of Interest

The authors declare that the research was conducted in the absence of any commercial or financial relationships that could be construed as a potential conflict of interest.
